# Supplementary material for: E-contact facilitated by conversational agents reduces interethnic prejudice and anxiety in Afghanistan
Source: Commun Psychol. 2024 Mar 21;2:22. doi: 10.1038/s44271-024-00070-z (PMC11332027; doi:10.1038/s44271-024-00070-z)
Supplement: Supplementary file 2 — Reporting Summary [file 44271_2024_70_MOESM2_ESM.pdf]

nature portfolio

Corresponding author(s): Sofia SAHAB

Last updated by author(s): Mar 1, 2024

Reporting Summary

Nature Portfolio wishes to improve the reproducibility of the work that we publish. This form provides structure for consistency and transparency in reporting. For further information on Nature Portfolio policies, see our Editorial Policies and the Editorial Policy Checklist.

Statistics

For all statistical analyses, confirm that the following items are present in the figure legend, table legend, main text, or Methods section.

n/a	Confirmed	
☐	☒	The exact sample size (*n*) for each experimental group/condition, given as a discrete number and unit of measurement
☐	☒	A statement on whether measurements were taken from distinct samples or whether the same sample was measured repeatedly
☐	☒	The statistical test(s) used AND whether they are one- or two-sided<br>*Only common tests should be described solely by name; describe more complex techniques in the Methods section.*
☒	☐	A description of all covariates tested
☐	☒	A description of any assumptions or corrections, such as tests of normality and adjustment for multiple comparisons
☐	☒	A full description of the statistical parameters including central tendency (e.g. means) or other basic estimates (e.g. regression coefficient) AND variation (e.g. standard deviation) or associated estimates of uncertainty (e.g. confidence intervals)
☐	☒	For null hypothesis testing, the test statistic (e.g. *F*, *t*, *r*) with confidence intervals, effect sizes, degrees of freedom and *P* value noted<br>*Give P values as exact values whenever suitable.*
☐	☒	For Bayesian analysis, information on the choice of priors and Markov chain Monte Carlo settings
☒	☐	For hierarchical and complex designs, identification of the appropriate level for tests and full reporting of outcomes
☐	☒	Estimates of effect sizes (e.g. Cohen's *d*, Pearson's *r*), indicating how they were calculated

Our web collection on statistics for biologists contains articles on many of the points above.

Software and code

Policy information about availability of computer code

Data collection	The study collected primary data on the demographic characteristics, intergroup prejudice, and intergroup anxiety of participants using SurveyMonkey, an online survey platform.
Data analysis	All code used for statistical analyses is available online at the link below.<br>https://osf.io/js5dq/?view_only=fcf0e3897425458abad2cb5b42d74318

For manuscripts utilizing custom algorithms or software that are central to the research but not yet described in published literature, software must be made available to editors and reviewers. We strongly encourage code deposition in a community repository (e.g. GitHub). See the Nature Portfolio guidelines for submitting code & software for further information.

Data

Policy information about availability of data

All manuscripts must include a data availability statement. This statement should provide the following information, where applicable:

- Accession codes, unique identifiers, or web links for publicly available datasets
- A description of any restrictions on data availability
- For clinical datasets or third party data, please ensure that the statement adheres to our policy

The dataset that underlies Table 2 is deposited and can be found at the link below.
https://osf.io/ydnwz/?view_only=9661e2571fae4ac6999ba129bf8d726a

Other data that support the findings of this study are not openly available due to reasons of sensitivity. The data are available from the corresponding author upon request. Requests for access will be reviewed within 30 days by the corresponding author. The data can be used only for academic research purposes via a data use agreement. Access, if granted, will be provided in a manner consistent with the original informed consent and privacy assurances given to study participants.

Human research participants

Policy information about studies involving human research participants and Sex and Gender in Research.

Reporting on sex and gender	Sex was considered in the study design as we randomly assigned participants to either all-female or all-male groups, which is common in contact/E-contact experiments based on Allport's Contact Hypothesis. However, sex was not analyzed as a variable in the study, and no sex-based analyses were performed. Information on the number of female and male participants within each ethnic group is provided in the Methods section. Sex was determined based on information provided by participants during registration for the experiment.
Population characteristics	See above.
Recruitment	The subjects were recruited through a respondent recruiting agency based in Afghanistan, which announced the call for participation on their online job portal as well as on their social networking sites. However, we acknowledge that self-selection bias may exist as individuals who visit the online job portal and choose to respond to the call for participation may differ from those who do not. To address this potential bias, we attempted to mitigate it by stratifying participants by ethnic group and randomly selecting from within each stratum during the recruitment process.
Ethics oversight	The experiments were conducted with the approval of the Ethics Committee of the Graduate School of Informatics, Kyoto University (KUIS-EAR-2021-020). Informed consent was obtained from participants at multiple points throughout the study.

Note that full information on the approval of the study protocol must also be provided in the manuscript.

Field-specific reporting

Please select the one below that is the best fit for your research. If you are not sure, read the appropriate sections before making your selection.

☐ Life sciences ☒ Behavioural & social sciences ☐ Ecological, evolutionary & environmental sciences

For a reference copy of the document with all sections, see nature.com/documents/nr-reporting-summary-flat.pdf

Behavioural & social sciences study design

All studies must disclose on these points even when the disclosure is negative.

Study description	The study utilized a randomized controlled trial (RCT) design to assess how CA facilitation influenced intergroup prejudice and anxiety. It employed a quantitative, longitudinal approach to track changes in these factors over time, both with and without the presence of a conversational agent.
Research sample	We recruited the subjects through a recruiting agency based in Afghanistan. The call for participation was announced by the agency on their portal as well as their social networking sites. Participants registered through SurveyMonkey. The procedure for selection of the subjects is described in Methods section (see also Fig. 1).
Sampling strategy	The study employed a stratified random sampling strategy, as described in the Methods section. An a priori power analysis was conducted using G*Power v3.1 to determine the appropriate sample size for a 2x3 ANOVA repeated measures, within-between interaction. Based on a small effect size (partial $\eta 2 = 0.02$, effect size $f = 0.14$), a significance level of 0.05, and a power of 0.95, the calculated sample size was 128 participants.
Data collection	The data collection procedure involved an online survey administered using SurveyMonkey, a web-based survey tool. Participants were able to complete the survey on their own computers or mobile devices. No one else was present during the data collection, and the researcher was not present either.
Timing	The data collection period for the study was from May 23, 2022, to June 9, 2022
Data exclusions	No data was excluded from the final analysis. However, before random selection, some participants (n=1795) were excluded due reasons described in Methods. The remaining 1,226 participants were divided into strata and randomly selected for the study.
Non-participation	No participants dropped out of the study.
Randomization	Stratified random sampling were used. Participants were randomly assigned to either control or treatment groups.

Reporting for specific materials, systems and methods

We require information from authors about some types of materials, experimental systems and methods used in many studies. Here, indicate whether each material, system or method listed is relevant to your study. If you are not sure if a list item applies to your research, read the appropriate section before selecting a response.

Materials & experimental systems

n/a	Involved in the study
☒	☐ Antibodies
☒	☐ Eukaryotic cell lines
☒	☐ Palaeontology and archaeology
☒	☐ Animals and other organisms
☒	☐ Clinical data
☒	☐ Dual use research of concern

Methods

n/a	Involved in the study
☒	☐ ChIP-seq
☒	☐ Flow cytometry
☒	☐ MRI-based neuroimaging
